# P-1630. Retrospective Evaluation of Urinalysis to Urine Culture Reflex Frequency and Criteria

**DOI:** 10.1093/ofid/ofae631.1796

**Published:** 2025-01-29

**Authors:** Dina Zheng, Elizabeth B Hirsch, Lina Hamid, Morgan L Bixby, Morgan L Bixby

**Affiliations:** University of Minnesota College of Pharmacy, Minneapolis, Minnesota; University of Minnesota College of Pharmacy, Minneapolis, Minnesota; M Health Fairview, Minneapolis, Minnesota; Univeristy of Minnesota, Saint Paul, Minnesota; Univeristy of Minnesota, Saint Paul, Minnesota

## Abstract

**Background:**

Patients with asymptomatic bacteriuria (ASB) often receive inappropriate treatment despite IDSA guidelines recommendation against antimicrobial treatment of ASB in the majority of adults. Emphasis on laboratory results rather than symptoms often triggers ASB treatment. At our institution, a urinalysis (UA) positive for ≥ 1 criteria ( > 10 WBC / HPF, leukocyte esterase (LE), or nitrites) results in a reflex to urine culture (UC). The objective of this study was to assess the frequency of UC reflex orders and the specific criteria driving reflex to identify opportunities for diagnostic stewardship and treatment guidance.

**Figure:**
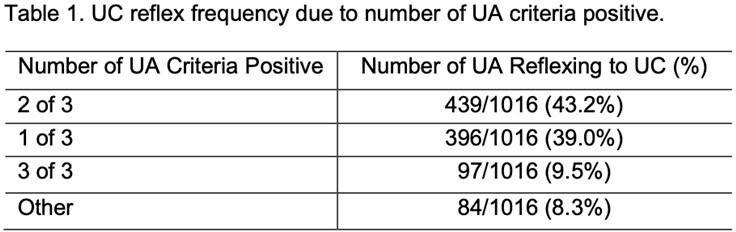

**Methods:**

Retrospective data were provided by the Best Practices Integrated Informatics Core for hospitalized adults with an ICD-10 diagnosis of urinary tract infection (UTI) or related condition and a UA +/- UC order within 72 hours between 11/1/2022 and 4/30/2023 at a large academic medical center. Number and frequency of UA to UC reflex and UA criteria driving UC reflex were assessed. LE results were considered positive if “moderate” or “large” and negative if “small”, “trace”, or “negative”. RStudio (version 2023.06.0) was utilized to perform logistic regression analyses with univariable and multivariable models to calculate odds ratios for reflex to UC.

**Figure:**
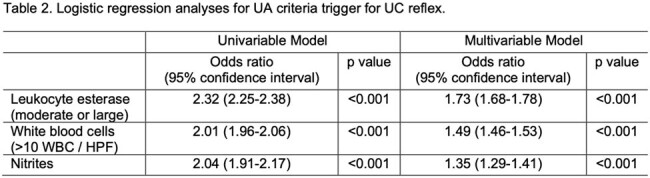

**Results:**

This cohort comprised 2007 unique patients with a total of 3193 UA orders. Of 3193 UA orders, 1016 (31.8%) reflexed to UC. The highest frequency of UC reflex was with 2 of 3 UA criteria positive (43.2%), followed by 1 of 3 criteria positive (39.0%), then 3 of 3 criteria positive (9.5%), and lastly, the remaining 8.3% of patients had UC reflexed for unknown reasons that need to be further investigated (Table 1). All 3 criteria when positive had increased odds for triggering UC reflex (Table 2). In the multivariable model, LE had the highest odds of triggering UC reflex (OR 1.73, 95% CI: 1.68-1.78).

**Conclusion:**

Despite frequent UA orders, only 31.8% reflexed to UC. Following these results, retrospective chart review will be completed to characterize patients with symptomatic UTI vs. ASB based on review of progress notes. These data will identify whether certain UA criteria are more highly correlated with symptomatic UTI and will help inform the need for revised UA and UC diagnostics.

**Disclosures:**

**Elizabeth B. Hirsch, PharmD, FCCP, FIDSA**, GSK: Advisor/Consultant|GSK: Honoraria

